# Measurement of optic nerve sheath diameter on computed tomography for the differentiation of transient ischemic attacks

**DOI:** 10.1590/1806-9282.20231001

**Published:** 2024-02-26

**Authors:** Rasime Pelin Kavak, Nezih Kavak, Senem Koca, Nurgül Balcı, Berna Turhan, Sümmeya Duran Kaymak

**Affiliations:** 1 Etlik City Hospital, Department of Radiology - Ankara, Turkey.; 2 Etlik City Hospital, Department of Emergency - Ankara, Turkey.; 3 The Republic of Türkiye Ministry of Health, General Directorate of Public Hospitals - Ankara, Turkey.

**Keywords:** Transient ischemic attack, Stroke, Optic nerve sheath, Computed tomography

## Abstract

**OBJECTIVE:**

The objective of this study was to investigate whether the measurement of mean optic nerve sheath diameter in patients with transient ischemic attack could be used to distinguish between control groups, the acute ischemic stroke group, and subgroups within the acute ischemic stroke category.

**METHODS:**

Retrospectively, the mean optic nerve sheath diameters of patients aged 18 years and older belonging to control, transient ischemic attack, acute ischemic stroke, and subgroups within the acute ischemic stroke category were measured with initial computed tomography conducted in the emergency department.

**RESULTS:**

Out of the 773 patients included in the study, 318 (41.1%) were in the control group, 77 (10%) had transient ischemic attack, and 378 (49%) were categorized as stroke patients. The average mean optic nerve sheath diameter was significantly higher in both the stroke and transient ischemic attack groups compared with the control group (p<0.001 for both comparisons). Furthermore, the mean optic nerve sheath diameter in the stroke subgroups was significantly higher than in both the transient ischemic attack and control groups (p<0.001 for all comparisons). In transient ischemic attack patients, the mean optic nerve sheath diameter showed a significant ability to predict transient ischemic attack (AUC=0.913, p<0.001), with a calculated optimal cutoff value of 4.72, sensitivity of 94.8%, and specificity of 73.9%.

**CONCLUSION:**

The mean optic nerve sheath diameter of patients in the transient ischemic attack group was lower compared with those in the stroke subgroups but higher compared with the control group.

## INTRODUCTION

Both acute ischemic stroke (AIS) and transient ischemic attack (TIA) arise from similar causes and exhibit comparable pathophysiological mechanisms. AIS is defined by the abrupt onset of neurological impairment caused by localized brain Ischemia and confirmed by imaging evidence of acute infarction. In contrast, TIA involves ischemic episodes with neurological deficits but without the presence of acute infarction^1^.

This is because TIA acts as an important indicator of an elevated risk of stroke. Without treatment, the risk of stroke within 3 months following a TIA can reach as high as 20%, with a significant portion of this risk occurring within the first 10 days, particularly within the initial 2 days^2^. It is essential for physicians to accurately differentiate between TIA mimics and potential TIA cases in order to initiate the appropriate treatment for patients. This distinction is of utmost importance not only to commence the correct therapy but also to avoid the potentially significant therapeutic implications that may arise from mistakenly initiating secondary prevention therapies, particularly in the elderly population^3^.

Noncontrast cranial computed tomography (CT) is the primary imaging modality of choice for assessing acute stroke symptoms in the ED. This preference stems from its widespread accessibility, cost-effectiveness, and rapidity^4^ ^,^ ^5^.

Following the principles of the Monroe-Kellie doctrine, the intracranial compartment can be defined as a closed system contained within the non-expandable rigid cranial structure. It comprises three non-compressible elements: brain tissue, blood, and cerebrospinal fluid. An elevation in intracranial pressure (ICP) can arise from an increase in one or more components of the intracranial compartment^6^. To maintain a normal ICP, any volume increase in one compartment must be counterbalanced by a subsequent reduction in volume within the other compartments. However, once these compensatory mechanisms are depleted, any additional increase in volume will result in significant elevations in ICP.

The optic nerve, a component of the central nervous system, is encompassed by cerebrospinal fluid and the dura mater, thus connecting it to the intracranial subarachnoid space. It is postulated that elevated ICP leads to the transmission of forces across these compartments, consequently causing enlargement of the optic nerve sheath. This phenomenon serves as an indirect indicator of alterations in ICP. As a result, the optic nerve sheath diameter (ONSD) has gained prominence as a non-invasive method for measuring increased ICP^7^.

The objective of this study was to investigate whether the measurement of mean ONSD in patients with TIA could be used to distinguish between control groups, the acute ischemic stroke group (AIS), and subgroups within the AIS category.

## METHODS

The study enrolled a consecutive series of adult patients (aged 18 years and above) who had undergone cranial CT examination for any reason in the Dışkapı Yıldırım Beyazıt Research and Training Hospital. Inclusion criteria encompassed patients who had not received a diagnosis of TIA or stroke (control group) and those diagnosed with TIA and AIS within 3 h from the onset of symptoms until the initial cranial CT in the emergency department (ED) between January 1, 2016, and December 1, 2022. Approval was granted by the Bilkent City Hospital Clinical Research Ethics Committee (Ankara, Türkiye) (ID number: 2023/3478).

The study assessed patient age, gender, final diagnoses (stroke, TIA), and the mean ONSD measurements. Exclusion criteria for all three groups included previous stroke, prior brain surgery, presence of ophthalmologic conditions that may affect optic nerve diameter, history of optic trauma, rheumatic and vasculitis syndromes, evidence of hemorrhage or mass lesions on cranial CT, artifacts in CT images obstructing visualization of the optic nerve, and patients receiving medical treatments that influence cerebrospinal fluid pressure. Patients in the control group were randomly selected from patients who underwent CT scanning for headache, syncope, and dizziness in the ED.

Patients were divided into three main groups: control, TIA, and stroke groups. Within the stroke group, further subgroups were established according to the Oxfordshire Community Stroke Project (OCSP) classification, including total anterior circulation infarct (TACI), partial anterior circulation infarct (PACI), posterior circulation infarct (POCI), and lacunar infarct (LACI)^8^.

### Computed tomography protocol and measurements

Computed tomography scans were conducted using a 64-detector row CT machine (GE Optima 660 SE 64 Detector 128-slice CT, General Electric Medical Systems, Milwaukee, WI, USA).

The ONSD measurements were obtained in millimeters at a distance of 3 mm behind the insertion of the optic nerve into the globe on the axial plane.

**Figure 1.**
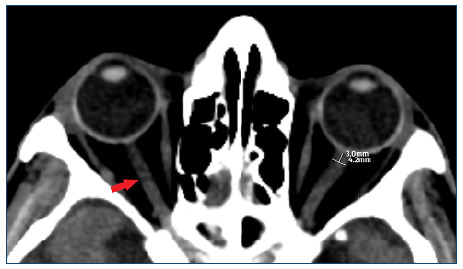
In the axial computed tomography section, the right optic nerves (red arrow) (a) and the location where the left optic nerve sheath diameter measurement was done (b).

The average ONSD value in millimeters, derived from the measurements of the optic nerve sheath of both the right and left eyes, was calculated for each patient and used as the mean ONSD value for further analysis. The measurements were performed using electronic calipers on the Extreme Picture Archiving and Communication System (ExtremePacs, Ankara, Turkey). All measurements were conducted by two experienced radiologists (R.P.K and B.T.) in a consensus in a blinded manner regarding the clinical data (Figure 1).

### Statistical method

Statistical analyses were performed using IBM SPSS Statistics for Windows, Version 20.0, IBM Corporation, Armonk, New York, USA. Descriptive statistics, including mean, standard deviation, median (interquartile range (IQR)) for continuous variables, and count and percentage for categorical variables, were reported. The normality of continuous variables was assessed using the Shapiro-Wilk test. Kruskal-Wallis variance analysis was utilized for comparisons of continuous variables among age groups. In cases where significant differences were observed following the Kruskal-Wallis variance analysis, multiple comparisons were conducted using the post-hoc test to determine the specific group differences. The chi-square test was employed for comparisons of nominal variables among groups (in cross-tabulations). The diagnostic accuracy of the mean optic nerve diameter values for TIA and stroke diagnoses was evaluated using the receiver operating characteristic (ROC) curves. The optimal cutoff point was determined using the Youden’s index. A significance level of p<0.05 was considered statistically significant.

## RESULTS

The mean age of the 773 patients included in the study was 54.53±11.69 (range: 18-77) years, with 368 (47.6%) being females. There was a statistically significant difference in age between the groups. Patients in the stroke group were older than those in the TIA group, and patients in the TIA group were older than those in the control group (for all comparisons, p<0.001).

There were no significant differences in the distribution of gender among the control, stroke, and TIA groups (p>0.05).

Among the patients, 318 (41%) were in the control group, 77 (10%) were diagnosed with TIA, and 378 (49%) were classified as stroke.

In the distribution of the stroke group by subtypes, LACI was the most common 110 cases (14.2%), followed by POCI infarct 93 cases (12%), PACI 89 cases (11.5%), TACI 86 cases (11.1%), and TIA 77 cases (10%), and in the control group, there were 318 cases (41.1%).

The mean ONSD values of the stroke and TIA groups were higher than those of the control group (p<0.001 for all comparisons) (Table 1).

**Table 1.** Comparison of control, stroke, and transient ischemic attack groups in terms of the mean optic nerve sheath diameter.

	Group 1: control	Group 2: stroke		Group 3: TIA		
	Mean±SD	Median	Mean±SD	Median	Mean±SD	Median
		(IQR)		(IQR)		(IQR)
Mean ONSD (mm)	4.58±0.24	4.5	5.68±0.45	5.55	5.03±0.20	5.1
		(4.4-4.7)		(5.4-5.8)		(4.8-5.2)
p-value	<0.001*	<0.001*		<0.001*		
Post hoc	Group 1-Group 2: **p<0.001**					
	Group 1-Group 3: **p<0.001**					
	Group 2-Group 3: **p=0.010**					

TIA: transient ischemic attack; ONSD: optic nerve sheath diameter. *Kruskal-Wallis analysis.

The mean ONSD of the control group was lower than that of the stroke subgroups and TIA group (p<0.001 for all comparisons). The mean ONSD of the TIA group was also lower than that of the stroke subgroups (p<0.001 for all comparisons) (Table 2).

**Table 2.** Comparison of the mean optic nerve sheath diameter values according to the stroke subgroups.

	Groups		Mean±SD	Median (IQR)	p-value*	Post hoc
Mean ONSD (mm)	Group 1	Control	4.58±0.24	4.5 (4.4-4.7)	**<0.001**	Group 1-Group 2: **p<0.001** Group 1-Group 3: **p<0.001** Group 1-Group 4: **p<0.001** Group 1-Group 5: **p<0.001** Group 1-Group 6: **p<0.001** Group 2-Group 3: **p=0.006** Group 2-Group 4: **p<0.001** Group 2-Group 5: **p<0.001** Group 2-Group 6: **p=0.002** Group 3-Group 4: **p=0.003** Group 3-Group 5: **p<0.001** Group 3-Group 6: **p<0.001** Group 4-Group 5: **p=0.004** Group 4-Group 6: **p<0.001** Group 5-Group 6: **p=0.002**
	Group 2	TACI	6.43±0.09	6.4 (6.3-6.5)		
	Group 3	PACI	5.70±0.12	5.7 (5.6-5.8)		
	Group 4	POCI	5.45±0.09	5.4 (5.4-5.5)		
	Group 5	LACI	5.28±0.16	5.3 (5.2-5.4)		
	Group 6	TIA	5.03±0.20	5.1 (4.8-5.2)		

TIA: transient ischemic attack; ONSD: optic nerve sheath diameter; TACI: total anterior circulation infarct; PACI: partial anterior circulation infarct; POCI: posterior circulation infarct; LACI: lacunar infarct. *Kruskal-Wallis analysis.

The mean ONSD also led to a significant prediction of TIA with an AUC of 0.913 (p<0.001). The optimal cutoff value was calculated as 4.72, with a sensitivity of 94.8% and specificity of 73.9%.

## DISCUSSION

Acute ischemic stroke and TIA represent critical conditions within the spectrum of cerebrovascular disorders and share common etiologies and pathophysiology^9^. This is the first study to establish a correlation between optic nerve sheath diameter measurements in the noncontrast cranial CT among control, TIA, AIS groups, and AIS subgroups. In this study, an increased ONSD diameter, indicative of elevated ICP, was observed in TIA patients compared with the control group, and this increase in ONSD was lower in acute stroke subgroups.

Various studies have reported normal mean ONSD values ranging from 3.68 to 4.99 mm in healthy individuals, whereas, in this study, the control group exhibited a mean ONSD of 4.58±0.24 mm^10^. The mean ONSD has no difference between age and gender^11^.

In the study of Komut et al.^12^, a significant difference in the mean ONSD between patients with stroke (5.4 mm) and the control group (4.1 mm) was observed. Similarly, Yüzbasıoğlu et al.^13^ reported a significant difference in the mean ONSD between stroke patients (5.6 mm) and the control group (3.6 mm). In this study, the mean ONSD in the TIA group was determined to be 5.03±0.20 mm. Furthermore, the mean ONSD in the stroke subgroups was higher than that of the TIA group, while the control group exhibited lower mean ONSD values compared with both the TIA and stroke subgroups.

In the study conducted by Gökcen et al.^14^, stroke patients were classified into four subgroups according to the OCSP classification. When comparing these subgroups with the control group in terms of right and left ONSD, a statistically significant difference was observed between all cerebrovascular disease subgroups and the control group. These findings indirectly suggest a significant increase in ICP across all cerebrovascular disease subgroups. Yavaşi et al.^15^ determined that the ONSD on cranial CT was higher in patients with ischemic AIS compared with those with TIA. Our results were similar.

It is recommended that the groups included in the study be homogeneous in terms of ONSD variations, age, gender, and previous diseases^16^. However, stroke is generally more common in older patients. The overall incidence of ischemic and hemorrhagic stroke has increased over the past decade to 85-94 per 100,000 in middle- and high-income countries and is much higher in people over 75 years of age (1151-1216 per 100,000)^17^. EDs consider adult patients from a wide range of age groups. As our study was retrospective, elderly patients admitted to the ED with stroke were included.

The ONSD measured using ultrasonography (USG) and CT were increased in patients with elevated ICP and the measurement of ONSD by USG and CT showed very high agreement^18^. Bhandari et al.^19^ analyzed alterations in ONSD through USG and CT both before and after ventriculoperitoneal shunt surgery in patients diagnosed with hydrocephalus. The researchers discovered a robust correlation between USG and CT scan measurements. While USG exams are generally more readily available than CT exams based on facility availability in healthcare centers, the operator of the equipment should be trained and ideally experienced in the area to make accurate measurements. As our study was retrospective in nature, measurements were obtained using CT results to explore the matter.

Reid et al.^20^ conducted a study using magnetic resonance imaging (MRI) arterial spin labeling sequence and observed regional subcortical or cortical hypoperfusion in patients with TIA compared with control subjects. Based on arterial spin labeling, the occurrence of lesions in patients with TIA is estimated to be between 35 and 56%. A study utilizing perfusion MRI with cerebral blood flow and time to maximum of the residue function maps demonstrated the presence of delayed perfusion or postischemic hyperperfusion in approximately one-fourth of TIA patients who had normal diffusion-weighted imaging findings. Lv et al.^21^ demonstrated that the resting-state blood oxygen level-dependent functional MRI with time-shift analysis could detect perfusion deficits in patients with TIA.

In our study, we assessed the mean ONSD in patients presenting within 3 h from symptom onset. Early-stage perfusion disturbances lead to an increase in ICP, followed by a subsequent decrease after reperfusion.

Additionally, Lakhani et al.^22^ reported a case series of three patients with TIA, highlighting the ability of 7T MRI to detect small areas of intracortical microhemorrhages and microinfarctions that may not be visible with lower field strengths. MRI or other standard scan methods used to diagnose TIA today may be insufficient to detect possible pathologies leading to ICP.

However, our study has some limitations, including being a single-site retrospective study design. Furthermore, none of our patients underwent invasive ICP monitoring.

## CONCLUSION

Cranial CT is the initially preferred method in the ED for evaluating patients suspected of having stroke or TIA. In this study, it was determined that the mean ONSD was lower in TIA subgroups of patients with stroke compared with stroke subgroups, but higher compared with the control group among patients who presented to the ED within the first 3 h.
